# Advancing Buntanetap: Comparative Pharmacokinetic Characterization of the Original Semi‐Crystalline with the Novel Crystalline Form in Animals and Humans

**DOI:** 10.1002/alz70861_108457

**Published:** 2025-12-23

**Authors:** Alexander Morin, Michael Christie, Maria L. Maccecchini

**Affiliations:** ^1^ Annovis Bio, Malvern, PA USA

## Abstract

**Background:**

Buntanetap is an orally bioavailable small molecule that improves cognitive function in patients with early Alzheimer’s and Parkinson’s diseases. Until now, a semi‐crystalline anhydrate (Form A) has been used in preclinical and clinical studies. However, recently a novel crystal dihydrate buntanetap (Form B) was discovered, offering improved solid‐state stability without compromising its bioavailability, metabolism and treatment efficacy. We present the pharmacokinetic (PK) profile of Forms A and B from bridge studies conducted in mice, dogs, and humans.

**Method:**

Form B was identified during a polymorph screen and structurally characterized by X‐Ray Powder Diffraction (XRPD). CD‐1 mice (*n* =6) received 50mg/kg buntanetap via oral gavage; plasma, CSF, and brain samples were collected at intervals up to 10h. Beagle dogs (*n* =12) were dosed orally with 20mg/kg, with plasma collected up to 12h post‐dose. Human volunteers (*n* =35) received a 50mg buntanetap capsule, and plasma was sampled up to 24h. Buntanetap levels were assessed by LC‐MS/MS and non‐compartmental analysis.

**Result:**

The two forms are distinct, do not interconvert, and exhibit comparable PK profiles across species. Forms A and B reach fast plasma Cmax (<2h), high brain/CSF and brain/plasma ratios, and complete systemic clearance by 12h post‐dose. Furthermore, both forms produce identical PK patterns for its primary metabolites, N1‐ and N8‐norbuntanetap, confirming that Form B retains the established metabolic characteristics of buntanetap.

**Conclusion:**

These findings support the continued development of Form B for future clinical use, as it combines enhanced solid‐state stability with a preserved PK profile essential for buntanetap’s therapeutic efficacy. Form B has also been approved by the FDA for all future clinical studies including the ongoing pivotal Phase 3 Early AD trial.

